# 45A ncRNA Expression Leads to Chromosomal Instability and Cytoskeletal Dynamics Impairment by Modulating GTSE1/p53/AurB Subcellular Localization

**DOI:** 10.3390/ijms27114892

**Published:** 2026-05-28

**Authors:** Matilde Calderoni, Silvia Viaggi, Paola Modesto, Tullio Florio, Aldo Pagano

**Affiliations:** 1 Department of Experimental Medicine, University of Genova, 16132 Genoa, Italy; calderoni.matilde@gmail.com; 2 DISTAV-Department of Earth, Environment and Life Sciences, University of Genoa, 16132 Genoa, Italy; silvia.viaggi@unige.it; 3 Laboratory of Human Genetics, IRCCS Istituto G. Gaslini, 16147 Genoa, Italy; 4 IRCCS Azienda Ospedaliera Metropolitana (IRCCS AOM), Plesso Ospedale Policlinico San Martino, 16132 Genoa, Italy; paola.modesto@hsanmartino.it (P.M.); tullio.florio@unige.it (T.F.); 5 Department of Internal Medicine (DIMI), University of Genoa, 16132 Genoa, Italy

**Keywords:** neuroblastoma, non-coding RNA, chromosomal instability

## Abstract

45A non-coding RNA overexpression induces modifications to the neuroblastoma cell cytoskeleton, leading to a cascade of reactions that interfere with proliferation control, cell migration, and tumorigenic potential. Through real-time RT-PCR, Western blotting, and immunofluorescence analysis, we investigated the different expression and/or localization of GTSE1, MCAK, Aurora B, and p53 and the altered organization of tubulin in different NB cell models stably overexpressing or downregulating 45A ncRNA. The proper regulation of these proteins’ expression and function is fundamental in cytoskeleton organization, as their impairment leads to chromosomal instability. We demonstrate that 45A ncRNA not only directly regulates the expression of the aforementioned proteins but can also affect GTSE1 subcellular localization: in 45A-overexpressing cells, the protein is accumulated in nuclei, while 45A downregulation leads to significant GTSE1 cytoplasm relocation and simultaneous p53 cytoplasmatic sequestration. This shuttling of the oncosuppressor reduces the apoptotic potential of 45A-downregulating cells, explaining the observed resistance to toxoids. Furthermore, 45A overexpression leads to an increased number of abnormal spindles, thus promoting chromosomal instability and possibly explaining the increased tumorigenic potential exhibited by 45A-overexpressing cells. These data highlight the role of 45A ncRNA in the functional regulation of several proteins involved in microtubule dynamics, supporting its possible relevance in prognosis.

## 1. Introduction

In the recent past, we discovered 45A, a pol III-transcribed non-coding RNA (ncRNA) that maps to intron 1 of the Amyloid Beta Precursor Protein-binding Family B member 2 (APBB2, FE65-like, FE65L1) gene in an antisense configuration. Its expression in neuroblastoma (NB) cells regulates several aspects of cytoskeleton functioning, determining phenotypical changes involved in cell proliferation, migration and adhesion, metastatic potential, tumorigenicity, and DNA repair efficiency. The effects of its overexpression/downregulation have been thoroughly analyzed in previous works. These articles also identified the overexpression of cycle-regulatory genes and downregulation of repair genes in the case of the overexpression of 45A, while the gene most downmodulated in the case of 45A silencing was GTSE1 [1,2,3]. In particular, we described the correlation between 45A expression and metastatic nodule localization/homing [2], suggesting that ncRNA expression is a key player in metastasis formation. Through amyloid beta precursor protein-binding family B member 2 (APBB2, FE65-like, FE65L1) alternative splicing, the expression level of 45A regulates the synthesis of G2 and S-Phase Expressed 1 (GTSE1), a protein involved in the regulation of cytoskeleton dynamics [3]. Indeed, GTSE1 can tune the microtubule (MT) stability of kinetochore–MTs by inhibiting the microtubule depolymerase mitotic centromere-associated kinesin (MCAK), triggering correct chromosome alignment and segregation [4]. Since MCAK regulates microtubule depolymerization, its dysregulation plays an important role in tumorigenesis, leading to the impairment of mitotic division and ultimately to chromosomal instability (CIN), a condition that contributes to the initiation of several types of tumor [5,6,7]. Furthermore, silencing of GTSE1 leads to hyperactivity in MCAK, causing kinetochore-associated microtubule destabilization, which leads to defects in chromosome alignment and spindle positioning [4,8,9]. On the contrary, a high expression of GTSE1 increases chromosome mis-segregation and CIN [3,4]. Thus, the inhibition of MCAK activity driven by GTSE1 regulates the balance of MT stability that, in turn, affects the fidelity of chromosome alignment, segregation, and stability [4]. In parallel, other groups have described the major importance of the MCAK-dependent regulation of centromere-associated microtubule dynamics in endothelial cell polarization and migration [10,11]. Besides the regulatory role of GTSE1, Aurora B (AurB) also plays an important role in the control of microtubule depolymerization through the regulation of MCAK activity [12,13,14]. This scenario clearly resembles the phenotype changes associated with microtubules, induced by the up-/downregulation of 45A ncRNA expression described above [1,2]. Moreover, the upregulation of GTSE1 and the enhancement of its regulatory activity is of particular interest in carcinogenesis. Indeed, it has been demonstrated that the upregulation of GTSE1 expression affects p53 subcellular localization, driving the protein from the nucleus to the cytoplasm, inhibiting its pro-apoptotic activity in cells undergoing carcinogenic transformation [15,16], and allowing cancer cells to survive independently of their primary lesion or oncogene activities [3,17,18,19]. In this context, 45A ncRNA, acting as an upstream regulator of GTSE1 expression and consequently of MCAK activity, is crucial in regulating CIN in cancer and is thus key in studying carcinogenesis in vivo.

In this work, we hypothesize that 45A expression levels may be prognostic for CIN in different patient/cancer types, and we investigate, at the chromosomal level, the effects of the modulation of its expression in neuroblastoma cells. In particular, we report that the modulation of 45A expression levels strongly affects GTSE1 subcellular localization and leads to the shuttling of p53 from the nucleus to the cytoplasm, lowering its pro-apoptotic activity. We also show that alterations to the 45A/GTSE1/MCAK/Aurora B-dependent axis not only leads to chromosome mis-segregation and impaired spindle organization and CIN but is also critical in the cell’s response to anticancer agents that target microtubule organization, confirming 45A ncRNA as a novel prognostic marker.

## 2. Results

### 2.1. The Modulation of 45A ncRNA Expression Drives GTSE1 Subcellular Localization

**Figure 1:**
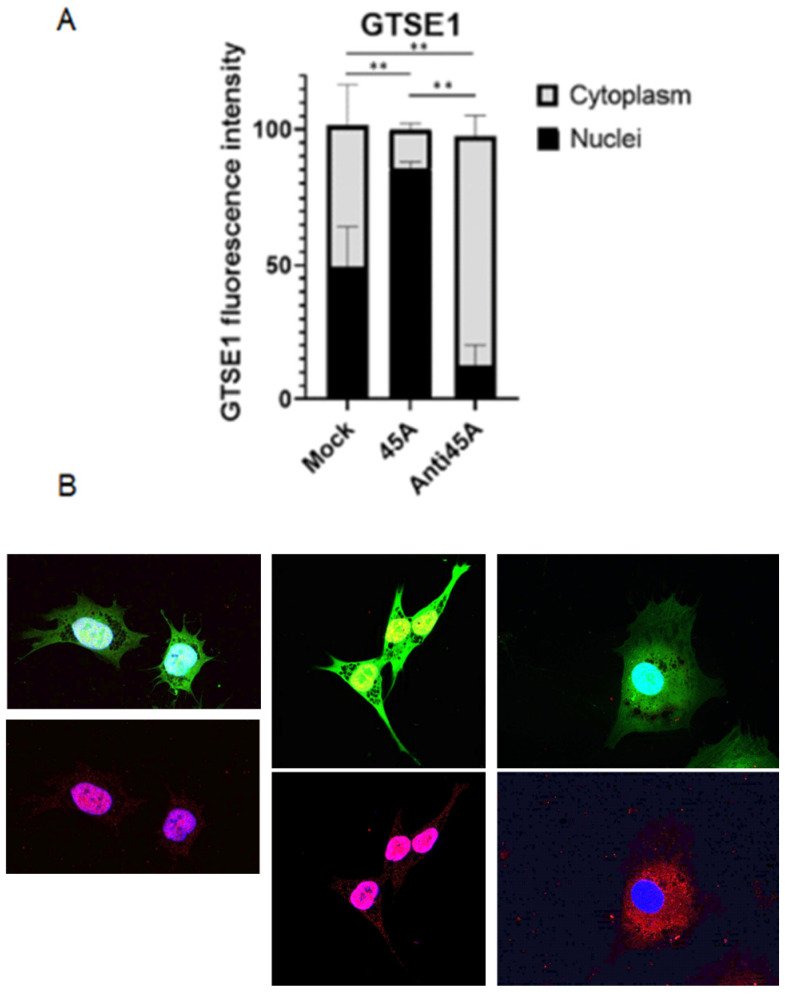
Subcellular localization of GTSE1. Quantification of GTSE1 (**A**) immunoreactivity in both the nucleus and cytoplasm in Mock, 45A, and Anti45A cells as a result of fluorescence quantitative analysis of 10 microscope fields. (**B**) Representative images of Mock, 45A, and Anti45A cells labeled with GTSE1 antibody: blue = Hoechst; red = GTSE1. Data represent mean ± SD; ** *p* < 0.01.

We previously demonstrated that changes in 45A ncRNA expression are paralleled by the modulation of GTSE1 expression via the alternative splicing of APBB2 [1]. However, not only are the quantitative changes in GTSE1 content relevant to its biological functions but variations in its subcellular localization may also determine its final activity. Indeed, the shuttling of GTSE1 between nucleus and cytosol drives the cytoplasmatic localization of p53, causing the loss of its pro-apoptotic activity [20,21]. To delve into this mechanism, we aimed to confirm whether the modulation of 45A expression could also determine a different subcellular localization of GTSE1 protein. To achieve this aim, we performed immunofluorescence (IF) with anti-GTSE1 antibodies on SKNBE2 neuroblastoma cells genetically engineered to express 45A ncRNA at physiological (Mock), overexpressed (45A), or downregulated (Anti45A) levels [1,2,3]. In this cell model, we measured the intensity of GTSE1 fluorescence in both the nucleus and cytoplasm and demonstrated that, compared to Mock cells, which displayed a homogenous distribution of GTSE1 (about 50% in the nucleus/cytosol), in 45A cells, GTSE1 was mainly localized to the nucleus (86%) and barely present in the cytoplasm (14%); moreover, in the Anti45A cells, in which GTSE1 expression was also significantly downregulated, it was mainly localized to the cytoplasm (85%) rather than the nucleus (15%) (Figure 1). These results demonstrate that the modulation of 45A ncRNA expression regulates not only GTSE1 synthesis [2,3] but also its subcellular localization.

### 2.2. 45A ncRNA/GTSE1 Axis Regulates p53 Nucleus/Cytoplasm Shuttling

**Figure 2:**
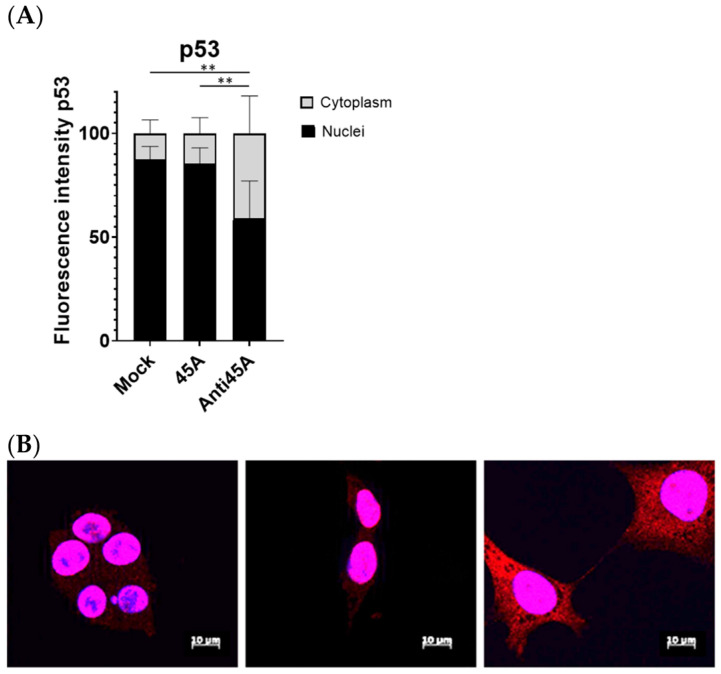
Subcellular localization of p53. Quantification of p53 (**A**) immunoreactivity both in nucleus and cytoplasm in Mock, 45A, and Anti45A cells as the result of the fluorescence quantitative analysis of 10 microscope fields. (**B**) Representative images of Mock, 45A, and Anti45A cells labeled with Anti-GTSE 1 antibody: blue = Hoechst; red = GTSE1 p53. Data represent mean ± SD; ** *p* < 0.01.

GTSE1 controls p53 at a transcriptional level and influences its subcellular localization [16,22]. Therefore, we hypothesized that 45A ncRNA, regulating GTSE1 expression and subcellular localization, may be an upstream regulator of p53 pro-apoptotic activity. To verify this hypothesis, we performed IF experiments using an anti-p53 antibody to quantify its localization in the nucleus and cytoplasm of Mock, 45A, and Anti45A cells. From the quantitative analysis of several microscope fields, we report that p53 is mainly located in the nucleus of cells that express 45A ncRNA and GTSE1 at endogenous (Mock cells) and overexpression (45A cells) levels, whereas its subcellular localization is equally distributed between the nucleus and the cytoplasm in cells where GTSE1 is downregulated (Figure 2). Given that the role of GTSE1 in binding and relocating p53 is already known in the literature [23], it is therefore significant that shuttling of the complex GTSE1/p53 occurs in the cytoplasm of Anti45A cells, showing that the overexpression of 45A ncRNA plays a key role in controlling p53 activity and, consequently, its pro-apoptotic response. In this context, it should be noted that the SKNBE cell line exhibits reduced tumor suppressor activity, as p53 is mutated, although the effects of this do not affect the phenomena described here.

Altogether, these results agree with our previous observation that the overexpression of 45A ncRNA confers susceptibility (whereas its downregulation confers resistance) to antimitotic and alkylating agents (adriamycin and methylmethansulfonate) that act in the nucleus and are not related to changes in microtubule dynamics. This 45A ncRNA-dependent mechanism is also associated with an increase in proliferation rate and a downregulation of genes involved in DNA repair and cell cycle checkpoint activation in response to DNA damage (i.e., GADD45A and HUS1) [1,2,3].

### 2.3. 45A Expression Modulation Impairs Cell Response to Anticancer Drugs That Target Microtubule Dynamics

**Figure 3:**
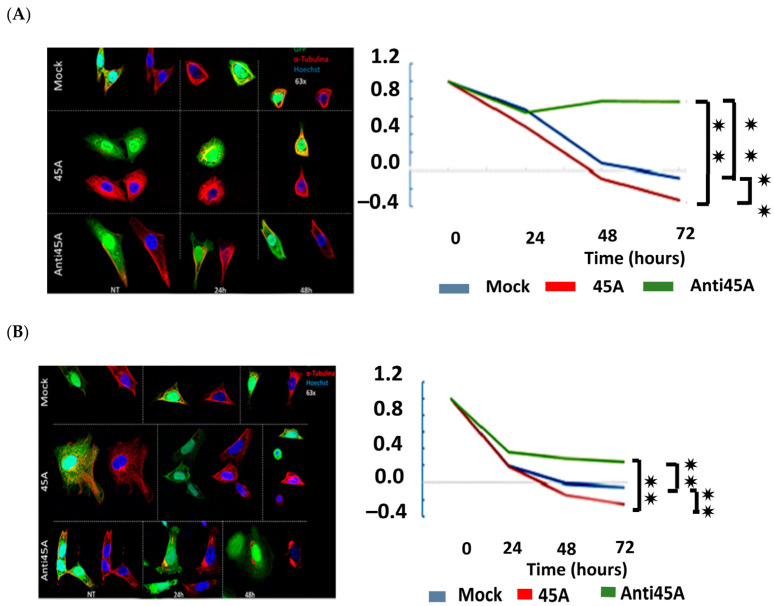
Evaluation of effects of mitotic spindle poisons on SKNBE2 Mock, 45A, and Anti45A cells. (**A**) Representative images of cytoskeleton organization during Paclitaxel treatment and drug response evaluated with xCELLigence system. (**B**) Representative images of cytoskeleton organization during vincristine treatment and drug response evaluated with xCELLigence system. ** *p* ≤ 0.01.

Alongside the induction of susceptibility to chemopharmaceuticals caused by the overexpression of 45A [1], we also investigated whether increased drug susceptibility can be induced by molecules acting on the polymerization/depolymerization dynamic of the cytoskeleton, dysregulated by the modulation of the 45A/APBB2/GTSE1 pathway. For this purpose, we treated Mock, 45A, and Anti45A cells with different concentrations (0.02, 0.2, 2.0 μM) of Paclitaxel (taxol), an inhibitor of microtubule polymerization, a process strongly influenced by the expression levels of 45A [1,2]. As shown in Figure 3A, Mock cells expressing endogenous levels of 45A are, as expected, susceptible to the action of Paclitaxel at a concentration of 0.2 μM starting from 24 h from the beginning of treatment, reaching a maximal inhibition at 72 h. 45A-overexpressing cells show a higher, time-dependent drug susceptibility. On the contrary, Anti45A cells do not display a reduction in cell survival after treatment with this concentration of Paclitaxel, indicating that the level of susceptibility of SKNBE2 cells to Paclitaxel is directly related to the level of impairment of cytoskeleton dynamics as a result of the deregulation of the 45A/APBB2/GTSE1 pathway (Figure 3A). Comparable results were obtained using two different concentrations (see Supplementary Figure S1). To strengthen these results, we also treated cells with vincristine, another drug that interferes with microtubule dynamics during proliferation. Results show that also this antitumor drug is mainly effective in 45A-overexpressing cells and encounters significantly increased resistance in cells that express a low level of 45A, confirming what we observed under Paclitaxel treatments (Figure 3B).

To evaluate changes in the cytoskeleton following treatment with Taxol or vincristine for 24 or 48 h, we analyzed the different cell lines by IF with anti-α-tubulin antibody. Theresults showed that cells expressed significant levels of 45A collapse after 48 h of treatment, displaying a disorganized microtubule distribution, while the Anti45A cells showed a regular microtubule distribution at the same time point (Figure 3A,B). The analysis of several microscope fields confirmed the robustness of these data (see Supplementary Figure S2).

Altogether, these data suggest that the expression level of 45A can predict possible responses to therapeutic agents acting on microtubule dynamics.

### 2.4. 45A ncRNA Overexpression Impairs Spindle Organization During Mitosis

45A ncRNA transcription modulation leads to significant modifications to cytoskeleton organization that, in turn, affect the adhesion and migration of neuroblastoma cells. These effects are correlated with alterations in the expression of several genes involved in cell cycle control, including GTSE1 [2]. In this context, we investigated the effects of different expression levels of 45A on mitotic division progression to determine its involvement in tumorigenesis and impact on CIN onset.

**Figure 4:**
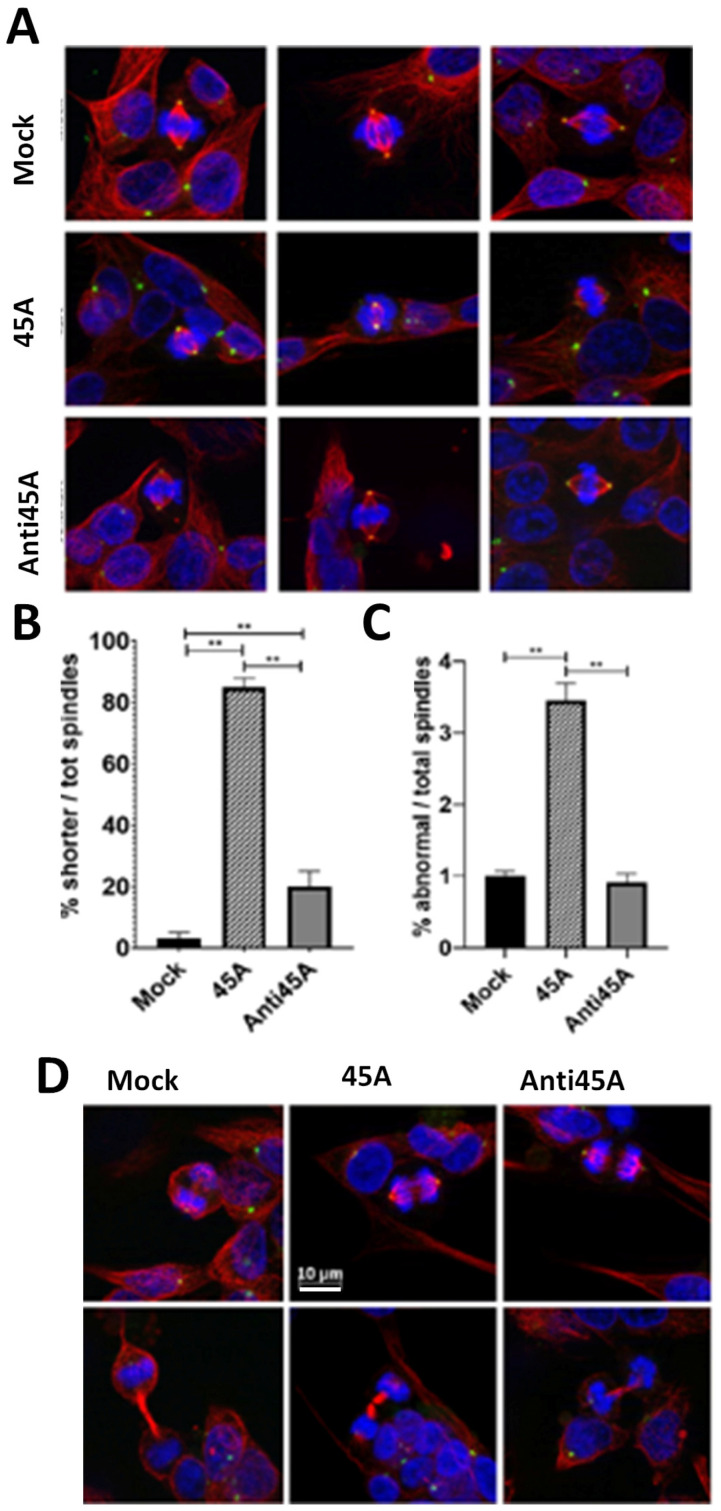
Centrosome and mitotic spindle immunofluorescence, representative images (**A**). Blue = Hoechst; red = α-tubulin; green = pericentrin. Distribution of short mitotic spindles (**B**) and of abnormal spindles (**C**) in the studied cell model. Data represent mean ± SD, ** *p* < 0.01. At least 100 mitotic figures were analyzed for each cell line (189 in Mock cells, 107 in 45A cells, 117 anti-45A cells, respectively) as the result of 4 independent experiments; (**D**) representative images of anaphase and telophase in SKNBE2 Mock, -45A, and -Anti45A cells. Blue = Hoechst; red = tubulin; green = pericentrin.

We performed immunofluorescence (IF) experiments with anti-α-tubulin and anti-pericentrin in SKNBE2 Mock, 45A, and Anti45A cells to detect mitotic spindle abnormalities and differences in mitotic phase distribution among the three clones. There was no difference in the percentage of cells in prophase, metaphase, anaphase, and telophase in 45A and Anti45A cells compared to Mock cells. These data were also confirmed via GIEMSA staining. On the contrary, IF staining showed a definite difference in the morphology and organization of the mitotic spindle: in 45A cells, we observed significant mitotic spindle shortening (Figure 4A,B) and a slight but significant increase in abnormal mitotic spindles (monopolar or multipolar) compared to SKNBE2-Mock and Anti45A cells (Figure 4C). Interestingly, we noted that in Anti45A cells, the interzonal fibers were missing during anaphase (Figure 4D); these are crucial elements in determining the location of contractile ring formation for cytokinesis. Significant differences were observed at the level of microtubule organization during cell division due to the different 45A expression levels.

### 2.5. MCAK Expression Is Not Directly Affected by 45A ncRNA Levels

GTSE1 plays a regulatory role in MCAK microtubule depolymerase activity [5]. Since 45A ncRNA regulates GTSE1 expression level and its subcellular localization, we hypothesized a role for 45A in the regulation of microtubule dynamics mechanisms. Therefore, we investigated the possible correlation between GTSE1 and MCAK expression and 45A content. To this aim, we performed IF analysis with anti-GTSE1 and anti-MCAK fluorescent antibodies on SKNBE2 Mock, 45A, and Anti45A cells.

**Figure 5:**
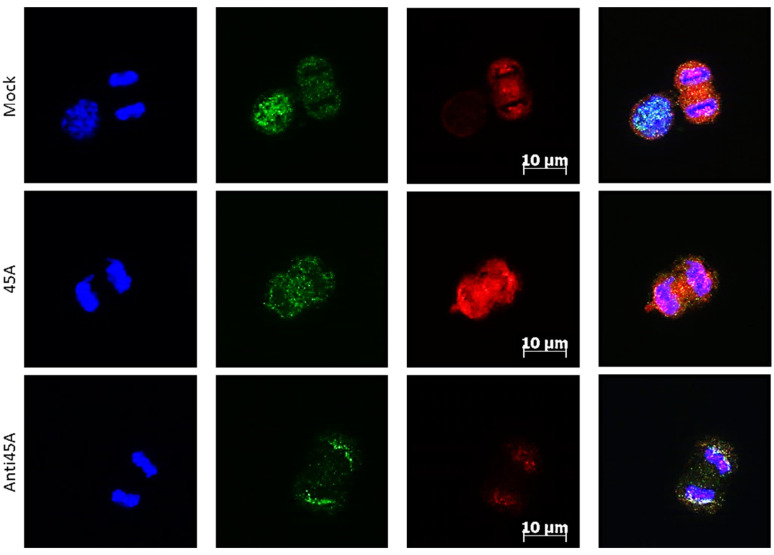
GTSE1 and MCAK immunofluorescence, representative images during anaphase. Blue = Hoechst; green = GTSE1; red = MCAK.

**Figure 6:**
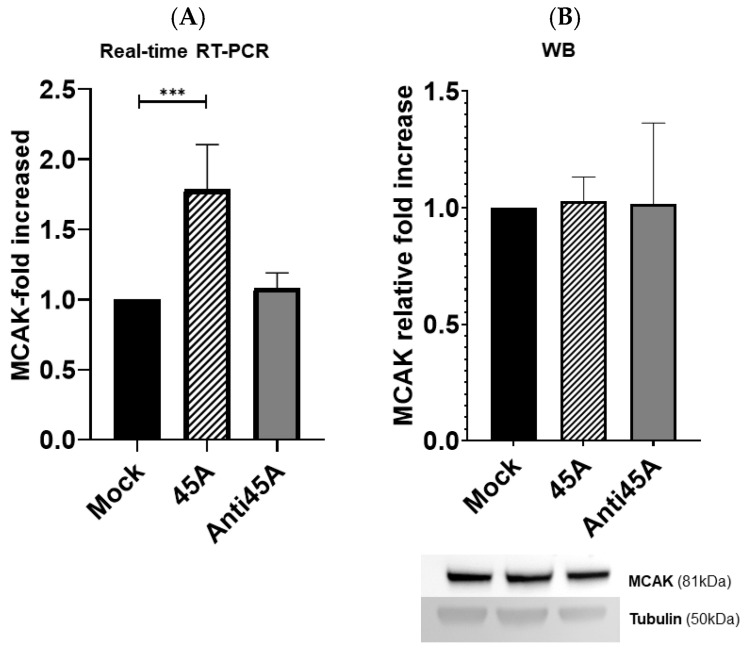
MCAK real-time RT-PCR (**A**) and Western blotting (**B**) analysis of Mock, 45A, and Anti45A cells. Data represent mean ± SD. *** *p* < 0.001 as the result of 4 independent experiments.

In preliminary experiments, we showed that the levels of 45A ncRNA do not affect the expression of MCAK during prophase and metaphase. On the contrary, during anaphase and telophase, GTSE1 and MCAK fluorescence signals were very low in Anti45A cells (Figure 5). Moreover, in Anti45A cells, GTSE1 and MCAK do not localize to the spindle midzone but remain bound to the chromosome centromeres (Figure 5). We used RT-PCR and WB to quantify MCAK mRNA and proteins in our cell models. As shown in Figure 6, although a powerful increase in mRNA was observed in 45A cells, there was no statistically significant modulation of MCAK protein expression in the three cell lines expressing 45A ncRNA at different levels, suggesting that GTSE1 may act by binding MCAK and regulating its activity at the post-translational level [6]. Altogether, these data indicate that MCAK does not represent a suitable molecular prognostic marker for neuroblastoma, although it could still represent a therapeutic target.

### 2.6. Downregulation of 45A Alters Aurora B Expression

In light of the tuned regulation of GTSE1, p53, and MCAK driven by 45A ncRNA expression level described above, we hypothesized a possible direct/indirect modulation of the activity of another key player of chromosome segregation control, the kinase Aurora B. Therefore, via IF staining, we analyzed the different subcellular localization of this protein during mitosis in SKNBE2 Mock, 45A, and Anti45A cells.

Aurora B kinase is the catalytic subunit of the chromosome passenger complex (CPC), which regulates many events in mitosis, including chromosome congression, kinetochore–microtubule attachments, spindle checkpoints, and chromosome segregation. It shows dynamic localization across the different mitotic phases. In particular, CPC is localized to the chromosome arms in prophase and then to the centromeres in prometaphase and metaphase. During anaphase, CPC and Aurora B are relocated to central spindle microtubules, and finally, in telophase, they are relocated to the midzone [24].

**Figure 7:**
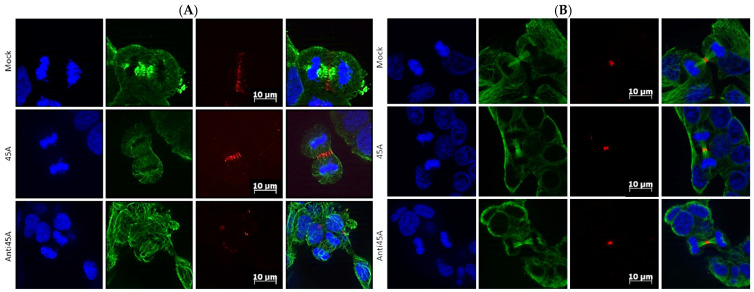
Cytoskeleton (**A**) and Aurora B (**B**) immunofluorescence, representative images of anaphase and telophase. Blue = Hoechst; green = β-tubulin; red = Aurora B.

Our results demonstrate that in both Mock and 45A cells, Aurora B is correctly localized in all mitotic phases, while in Anti45A cells, the Aurora B signal is almost undetectable during anaphase, reappearing in telophase (Figure 7). This can be explained by Aurora B being able to resume its activity thanks to the change in balance between dynamic and stable microtubules typical of these phases [8]. In this regard, it should be noted that our data do not exclusively demonstrate degradation; therefore, the protein could be diffused into the cytoplasm and then reappear in telophase.

**Figure 8:**
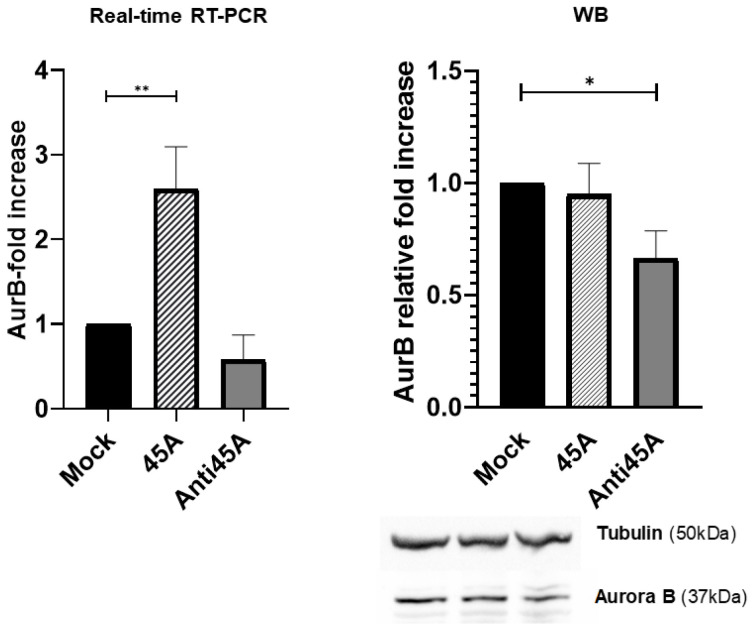
Aurora B Real-time RT-PCR and Western blotting analysis of Mock, 45A, and Anti45A cells. Equal loading of proteins was ensured by tubulin expression. Data represent mean ± SD; ** *p* < 0.01 as the result of 4 independent experiments. * *p* ≤ 0.05.

We then used RT-PCR and Western blotting to measure the expression levels of AurB mRNA and protein. We found that, at a transcriptional level, Aurora B is overexpressed in 45A cells, while there are no significant transcriptional differences between Anti45A and Mock cells. Interestingly, the quantification of Aurora B protein content demonstrated a significant downregulation in Anti45A cells (Figure 8). Altogether, these results underline the major impact of the 45A/GTSE1 pathway on cytoskeleton organization during mitosis.

### 2.7. GTSE1 and AurB Expression Levels Correlate with Prognosis in NB Patients

**Figure 9:**
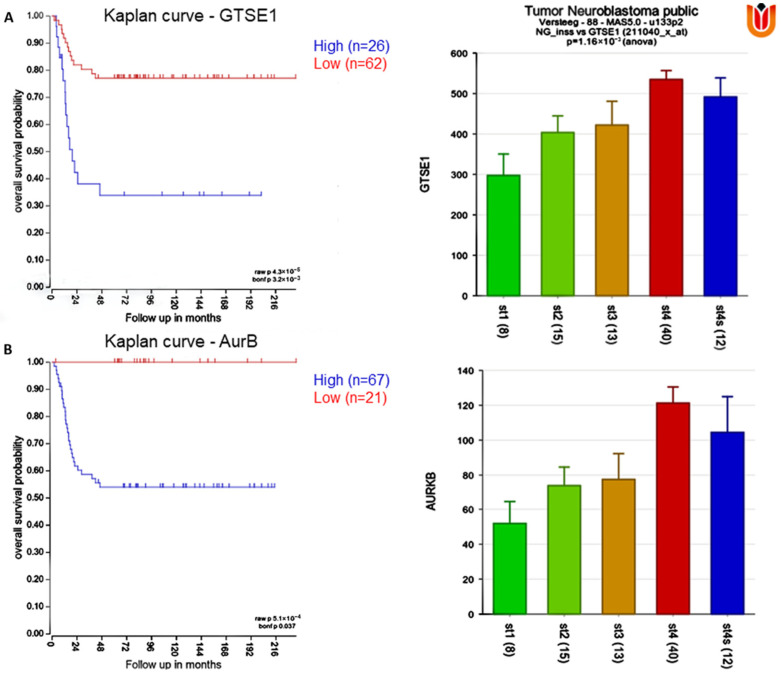
The panel shows the Kaplan–Meier results for 88 NB patients divided into two clusters (**A**). The curves for GTSE1 and AurB are shown at the top and bottom, respectively. Curves are relative to the patient’s overall survival, expressed in years. Red and blue curves represent good- and poor-prognosis patients, respectively. (**B**) Expression of GTSE1 (**top**) and AurB (**bottom**) in patients divided by NB stage. Images are taken from the R2 Neuroblastoma dataset.

Since 45A ncRNA-dependent control of GTSE1 expression exerts remarkable effects in several processes (i.e., DNA repair, cell adhesion and metastasis homing, CIN development, and susceptibility to anticancer compounds acting on cytoskeleton dynamics), we performed a univariate survival analysis using one publicly available gene expression dataset including 88 NB patients [25] to assess the possible correlation between GTSE1 and/or Aurora B expression and NB prognosis. We found that both GTSE1 and Aurora B expression were endowed with a significant prognostic value in terms of overall survival (*p* ** < 0.01) (Figure 9A). Notably, the level of expression of both GTSE1 and Aurora B also correlated with NB stages and the risk of succumbing to the disease, being more expressed in high-risk stages (Figure 9B). Altogether, these results highlight that GTSE1 and Aurora B may represent novel prognostic markers for NB and, following proper standardization, suitable tools in NB prognosis and staging.

## 3. Discussion

In previous works, we showed that the upregulation of 45A ncRNA transcription leads to the impairment of DNA repair and to specific cell phenotype alterations associated with tumorigenesis [1,2]. In this work, we highlight the significant role played by 45A ncRNA in controlling cytoskeleton dynamics during mitosis. Mitotic cells need to finely regulate the dynamics of their spindle microtubules and, in particular, those attached to chromosomes via kinetochores. Kinetochore microtubules stably attach to chromosomes and align them at the equator of the spindle, although the stability of this bond should still allow the correction of eventual attachment errors [26]. Cancer cells often have hyper-stable kinetochore microtubules, a condition that leads to an increase in attachment errors and chromosome mis-segregation. In turn, this causes cancer cells to gain or lose chromosomes, a phenomenon known as chromosome instability (CIN) [26].

45A ncRNA participates in the onset of CIN in NB cells due to its ability to influence the activity and/or synthesis of proteins essential in the control of microtubule dynamics during cell division. In particular, previous work in our laboratory demonstrated that GTSE1 protein synthesis is directly regulated by the 45A ncRNA transcription level. Moreover, the modulation of 45A ncRNA expression also affects the localization of GTSE1, as 45A-overexpressing cells accumulate GTSE1 in nuclei, whereas in Anti45A, the protein is also localized to the cytosol. In agreement with the observation that GTSE1 is a regulator of transcription and subcellular localization for p53 [16,17], our experiments show that the displacement of GTSE1 to the cytosol in Anti45A cells also causes p53 shuttling in the cytosol, where it is degraded. In this way, p53 pro-apoptotic activity is inhibited, accounting for the observed resistance to spindle poisons in cells in which 45A is downregulated.

Moreover, the reduction in Aurora B protein levels that we found in 45A-downregulated cells may also account for the development of drug resistance, in agreement with data published in non-small cell lung cancer cells [27]. In detail, in anti45A cells, we observed that Aurora B is missed during the anaphase, a possible consequence of the lack of GTSE1 activity, as it is downregulated in these cells; on the contrary, Aurora B is present at the midbody during telophase, where the changes in stable–dynamic microtubule ratio can restore its activity [10].

Rondelet et al. [28] reported that in U2OS osteosarcoma, depletion of GTSE1 in cells did not lead to any detectable decrease in Aurora B levels in mitotic cells or in its activity (as measured by quantifying histone H3 pSer10 on chromosomes). Together, these data indicate that defects in Aurora B level/activity, PEFs, or CENP-E function are unlikely to represent the cause of the chromosome misalignment observed following GTSE1 perturbation [28].

To further explore the role of GTSE1 in regulating MCAK microtubule depolymerization activity, we analyzed the expression of these two proteins in the neuroblastoma 45A-cell model. Although the precise mechanism underlying MCAK regulation by GTSE1 has not been completely defined, it has been proposed that the level of MCAK phosphorylation may be a relevant factor [6,29]. Interestingly, Bendre et al. [4] reported that the depletion of GTSE1 in U2OS cells enhances the depolymerization activity of MCAK, leading to defects in mitotic spindle organization and confirming the role of MCAK in regulating GTSE1.

Here, we demonstrate that different levels of GTSE1 (driven by 45A ncRNA expression levels) did not affect the synthesis of MCAK protein according to its regulation at the post-translational level. Alternatively, the degradation of the synthesized protein could be enhanced, thus masking an increase in its synthesis.

As well as highlighting the regulation of microtubule dynamics by the 45A/GTSE1/MCAK axis, our experiments also identified another primary actor in the regulation of microtubule organization in the mitotic spindle: Aurora B.

Fuller et al. [29] revealed a phosphorylation gradient in anaphase cells between the poles and the spindle midzone, where it culminated. Furthermore, they showed that the maintenance of AurB activity through interaction with microtubules at the midzone is required for the establishment of the gradient from which the cleavage furrow position can be predicted. In this context, although we lack a mechanistic explanation, we can report that alongside the undetectable presence of interzonal fibers in anaphase in Anti45A cells, our observations relating to GTSE1, MCAK, and AurB are in the same direction.

We also show that this protein is influenced by 45A/GTSE1 cellular contents in accordance with the mitotic spindle alterations occurring following their modulation. Notably, these data also account for the different responses of cells to cytotoxic drugs directed against components of microtubule organization.

The importance of this 45A-dependent regulatory pathway of cytoskeletal organization and subcellular localization of p53 in cancer cells was also demonstrated in a cohort of 88 individuals whose gene expression profile and survival rate were known. Moreover, in this case, we have brought to light a correlation between GTSE1 and Aurora B expression under 45A regulation and patient survival and staging.

## 4. Materials and Methods

### 4.1. Cell Cultures

SKNBE2 neuroblastoma cells were provided by the cell bank of the National Institute of Cancer Research (IST), Genoa, Italy, and obtained from ECACC. SKNBE2 (KCB Cat# KCB 201199YJ, RRID: CVCL_0528) and derived SKNBE2 cells were maintained on RPMI 1640 medium (ECB9006L EuroClone, Milan, Italy), 10% FBS (Euro Clone, Milan, Italy), L-glutamine (2 mM; EuroClone, Milan, Italy), and penicillin–streptomycin (100 U/mL/100 μg/mL; Euro Clone, Milan, Italy) (standard medium). Cells were selected in 200 μg/mL G-418 (Geneticin; Invitrogen, Monza, Italy) under standard culture conditions.

### 4.2. Immunofluorescence

Cells were cultured directly on coverslips of glass coated with poly-lysine. Cells were fixed with methanol: acetone (1:1) for 10 min at −20 °C, followed by 10 min methanol at −20 °C, and were then blocked with 3% BSA bovine serum albumin (BSA) containing 0.1% Triton X-100.

The following primary antibodies (and dilution) were used:

- Rabbit anti-α-tubulin (Abcam, Cambridge, UK) 1:300;
- Mouse anti-α-tubulin (Sigma-Aldrich, Saint Louis, MO, USA) 1:1000;
- Mouse anti-Aurora B (Abcam, Cambridge, UK) 1:50;
- Mouse anti-p53 (Santa Cruz Biotechnology, Dallas, TX, USA) 1:200;
- Mouse anti-MCAK (Santa Cruz, USA) 1:50;
- Rabbit anti-GTSE1 (Abcam, Cambridge, UK) 1:50;
- Rabbit anti-pericentrin (Abcam, Cambridge, UK) 1:500;
- Rabbit anti-APBB2 (Sigma-Aldrich, USA) 1:1000;
- Rabbit anti-fibrillarin (Santa Cruz, USA) 1:200.

After 1 h incubation with the antibodies diluted in PBS/1% BSA at 37 °C and 3 × 5 min washing in PBS, the cells were incubated with the secondary antibodies Alexa Fluor 568 goat anti-rabbit IgG and Alexa Fluor 594 goat anti-mouse IgG (Invitrogen, Monza, Italy), used at 1:500. The coverslips were then incubated with Hoechst 33342 to detect nucleus counterstaining.

Images were acquired with a Zeiss Axiovert 200 M (Zeiss, Milano, Italy) inverted microscope equipped with the ApoTome slide module (Zeiss, Milano, Italy) through a ×63 objective and processed using Zeiss AxioVision 4.8 software (Zeiss, Milano, Italy).

### 4.3. Quantification of Fluorescence Intensity

To determine the fluorescence levels from the microscopic images, we used the tool Analyze → Measure in ImageJ software v. 1.54p. After selecting the regions of interest in the image and the parameters to be measured (Analyse → Set Measurements → Area, Integrated density, mean gray value), the correct total fluorescence intensity (CTCF) was calculated, both in the whole cell and in the nucleus only, with the following formula:

CTCF = Integrated density − (Area of selected cell × Mean fluorescence of background area)

The background was obtained by selecting a region of the image without fluorescence. Finally, the fluorescence intensity in the cytoplasm alone was calculated by subtracting the CTCF in the nucleus from the total CTCF.

### 4.4. Quantitative Real-Time RT-PCR Analysis

Total RNAs from samples were extracted using TRIzol reagent (Invitrogen, Carlsbad, CA, USA) according to the manufacturer’s protocol and subjected to reverse transcription using the Transcriptor First-Strand cDNA Synthesis Kit (Roche, Mannheim, Germany), with a random hexamer, following the manufacturer’s instructions. The total RNA from samples was measured via real-time quantitative RT-PCR using the ABI PRISM^®^ 7700 Sequence Detection System (Perkin Elmer Corp./Applied Biosystems, Foster City, CA, USA) and the SYBR Green method following the manufacturer’s instructions.

The forward and reverse primer sequences used were as follows: 45A_F 5′-CATCTATAATGGCTGAATTGGAA-3′ and 45A_R 5′-ATGAACTTTCCAACAAATGTTGTT-3′; Aurora B_F 5′-AGAAGGAGAACTCCTACCCCT-3′ and Aurora B_R 5′-CGCGTTAAGATGTCGGGTG-3′; MCAK_F 5′-CTGTTTCCCGGTCTCGCTATC-3′ and MCAK_R 5′-TCTGGGTTTATTGCAGCCACA-3′.

For the endogenous control, the expression of the glyceraldehyde 3 phosphate dehydrogenase (GAPDH) gene was examined. The sequences for human GAPDH primers were 5′-GAAGGTGAAGGTCGGAGTC-3′ and 5′-GAAGATGGTGATGGGATTTC-3′. Relative transcript levels were determined from the relative standard curve constructed from stock cDNA dilutions and were divided by the target quantity of the calibrator, following the manufacturer’s instructions.

### 4.5. Western Blotting

Proteins were extracted from cells using RIPA buffer and a cocktail of protease and phosphatase inhibitors (Roche). Samples were then clarified via centrifugation at 12,000× *g* for 10 min at 4 °C and the supernatant recovered. Protein concentrations were determined using the colorimetric Bradford assay (Bio-Rad, Segrate (MI) Italy).

Western blotting was performed with the SDS-PAGE Electrophoresis System. Protein samples were electrophoresed on 4–12% Bis-Tris polyacrylamide gels (NP0322, Thermo Fisher, Monza, Italy) under reducing conditions and blotted to a nitrocellulose membrane (Whatman, Sigma-Aldrich Saint Louis, MO, USA). After the transfer, membranes were blocked with 5% non-fat dry milk for 1 h at room temperature and incubated overnight at 4 °C with the following primary antibodies: mouse anti-Aurora B (Abcam, Cambridge, UK) 1:1000, mouse anti-MCAK (sc-81305, Santa Cruz Biotechnology, Dallas, TX, USA), and mouse anti-α-tubulin (Sigma-Aldrich, USA) 1:4000. After three washes with TBST, membranes were incubated for 1 h at room temperature with goat anti-mouse IgG (Fc specific)–Peroxidase antibody (1:12,000, A0168 Sigma-Aldrich, USA) or goat anti-rabbit IgG Peroxidase antibody (1:16,000, A0545 Sigma-Aldrich). Bands were revealed with the ECL chemiluminescence detection system (Thermo Fisher, Monza, Italy). The densitometric analysis of protein bands was performed using the ImageJ software system v. 1.54p.

### 4.6. xCELLigence System Cytotoxicity Assays

Cell cytotoxicity was evaluated in real time by measuring electrical impedance across interdigitated gold micro-electrodes integrated at the bottom of tissue culture plates using the xCELLigence RTCA MP System (Roche, Mannheim, Germany). Cell–sensor impedance is expressed as an arbitrary unit called Cell Index.

SKNBE2 cells were seeded (3 × 10^3^ cells per well) in standard medium in E-plates (Roche), and compounds were added after 24 h. Different concentrations of Paclitaxel (TEVA) were tested (2, 0.2, and 0.02 um).

### 4.7. Gene Expression Dataset

One publicly available dataset was used for gene expression analysis. It contains the gene expression profile of 88 tumors measured using the Affymetrix 18 Human Genome U133 Plus 2.0 platform (GSE16476). The database is called “R2: Genomics Analysis and Visualization Platform”.

### 4.8. Statistical Analysis

Results are expressed as mean ± Standard Deviation. The statistical significance of observed differences among different experimental groups was calculated using one-way ANOVA with post hoc Tukey HSD. A *p*-value of less than 0.05 was considered statistically significant. In the figures, * and ** indicate statistical significance at *p* < 0.05 and 0.01, respectively. The statistical calculations were performed with GraphPad Prism 8.0 for Windows.

## 5. Conclusions

In summary, we currently know that 45A ncRNA affects APBB2 splicing, although the ways in which different protein variants can affect GTSE1 overexpression remain to be clarified and are currently under investigation. We have shown that increased GTSE1 levels have important effects on MCAK, Aurora B, and microtubule dynamics in NB cells and possibly in cancer cells more generally. In this scenario, therefore, the expression level of 45A ncRNA/GTSE1 could represent a prognostic tool for CIN in different types of cancers and/or predict response to some chemotherapeutics.

## Data Availability

The original contributions presented in this study are included in the article/Supplementary Material. Further inquiries can be directed to the corresponding authors.

## Supplementary Materials

The following supporting information can be downloaded at: https://www.mdpi.com/article/10.3390/ijms27114892/s1.

## Abbreviations

The following abbreviations are used in this manuscript:

MT	Microtubule
CIN	Chromosomal Instability
CTCF	Correct total fluorescence intensity
