# Supplementary figures and images for: 45A ncRNA Expression Leads to Chromosomal Instability and Cytoskeletal Dynamics Impairment by Modulating GTSE1/p53/AurB Subcellular Localization
Source: Int J Mol Sci. 2026 May 28;27(11):4892. doi: 10.3390/ijms27114892 (PMC13256316; doi:10.3390/ijms27114892)

### Paclitaxel 2uM

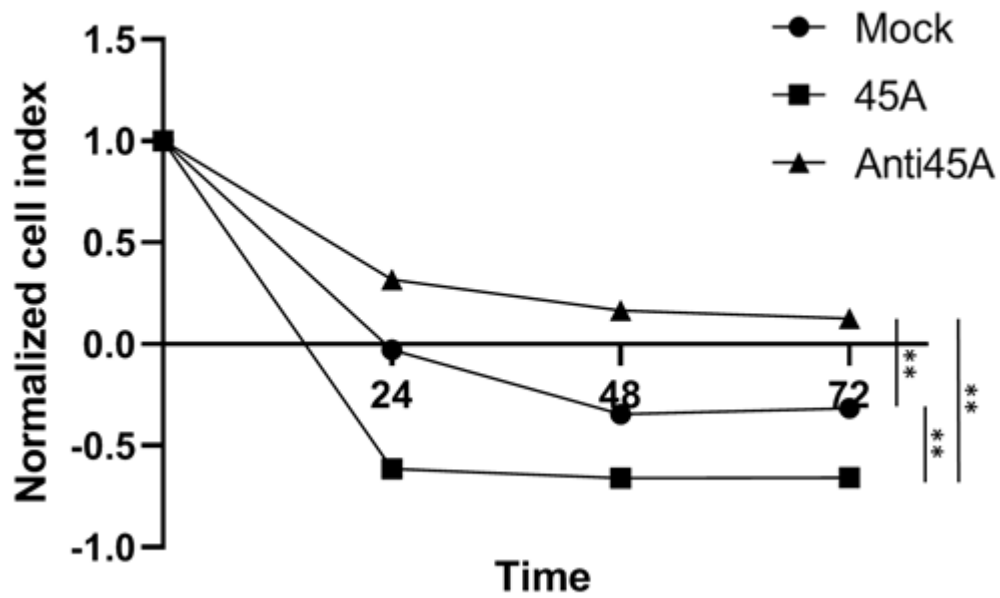

### Paclitaxel 0,02uM

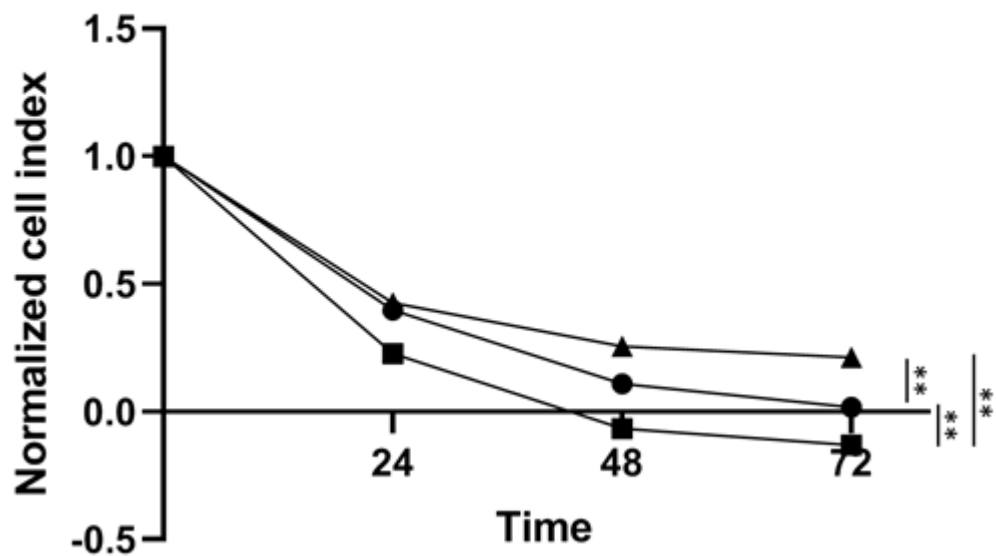

Supplement: Supplementary file 1 [file ijms-27-04892-s001.zip › Supplementary Figure S1.pdf]

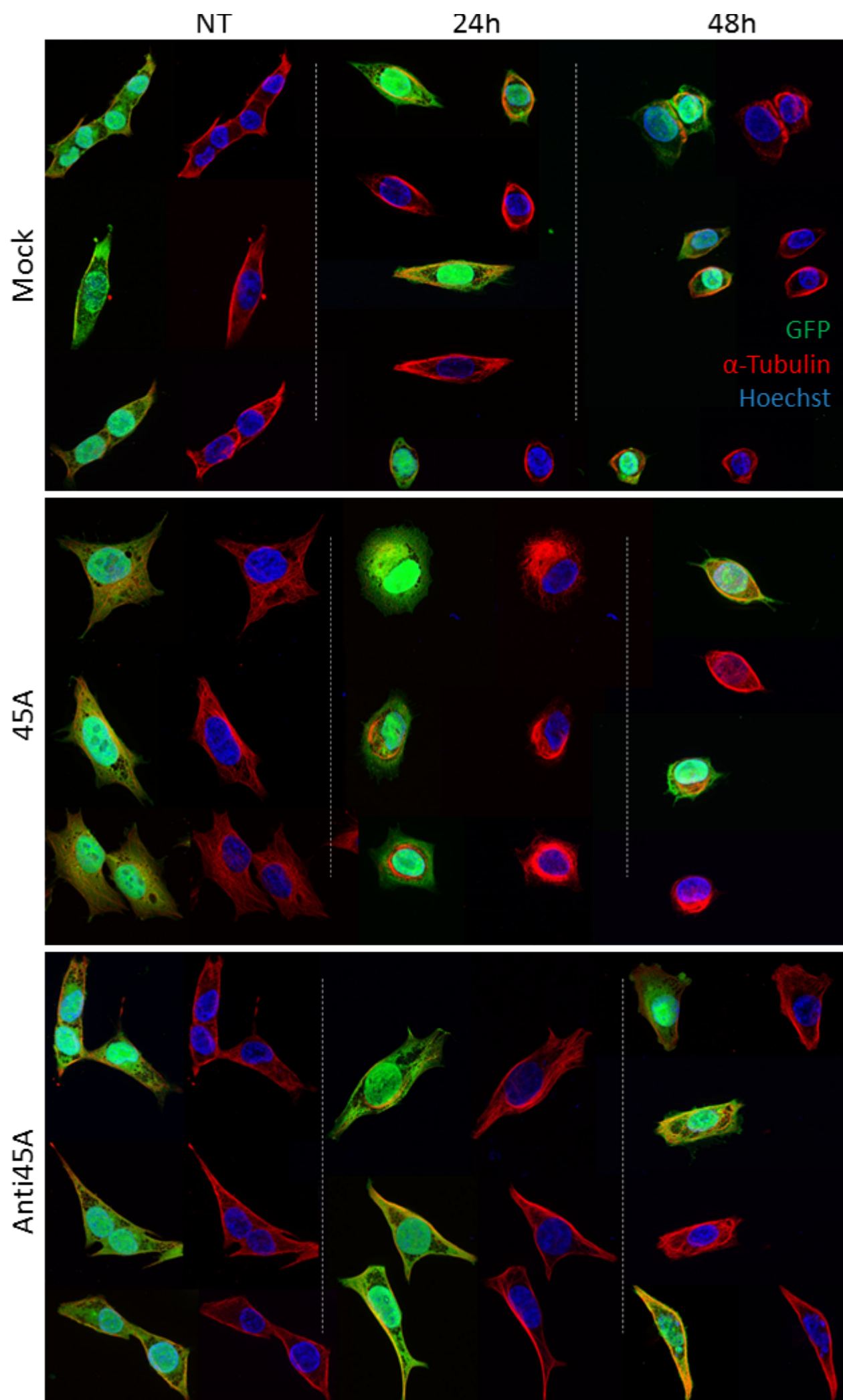

Supplement: Supplementary file 1 [file ijms-27-04892-s001.zip › Supplementary Figure S2.pdf]
